# How to Deliver Integrated Care

**DOI:** 10.5334/ijic.6221

**Published:** 2021-08-25

**Authors:** Wilma van der Vlegel-Brouwer, Robin Miller

**Affiliations:** 1 Onderzoek en advies, Nieuwerkerk aan den IJssel, The Netherlands; 2 Department of Social Work & Social Care, University of Birmingham, UK

**Keywords:** integration, management, leadership, social dimensions, values, implementation

## Abstract

Axel Kaehne, Henk Nies, Bingley: Emerald Publishing Limited, 2021, ISBN 9781838675295.

The European Health Management in Transition book series seeks to tackle “all the ‘disruptive’ changes that are re-framing the health way management needs to develop within health systems and organisations” (pxxi). This volume, which is edited by Axel Kaehne and Henk Nies, focusses on the management contribution to integrated care. A total of 25 authors around the globe provide practical solutions to common problems and dilemmas faced by health and care systems seeking to become more person centred and coordinated. After an initial introduction to the meaning and purpose of integrated care, the book consists of eight chapters which deals with a distinct domain of change.

The introductory chapter defines Integrated Care based upon the framework of Valentijn et al (2013). Beside the functional dimensions of integration on different levels, the chapter emphasises that care should be aligned with what a patient or user values most and this principle is reflected throughout the book. Chapter 2 outlines how integrated care initiatives can be funded through a framework based on the possible integrators, the existing payment models, and additional funding models. Chapter 3 on leadership considers frameworks which reflect leadership behaviours to support integration and develop supportive organisational cultures. It underlines that leadership is not only needed in formal positions, but on every level of a health and care system including by frontline professionals.

Engaging patients in the process of planning and delivering of integrated care services is the focus of Chapter 4. The principles of patient involvement and design thinking are brought to life through a fascinating case study from Queensland. The next two chapters consider the dynamics of collaboration between different stakeholders and professionals. Chapter 5 discusses the “social dimensions of care integration” and highlights that professionals have their own competencies, resources, interests and legislative space. They will therefore need to be guided by managers to move away from competition or power differentials and enhance their motivation by hearing everyones’ voices. Although main objectives and desires of professionals and programmes are often driven by the same values, these values can also be in conflict. Chapter 6 provides practical steps and exercises on how common ground and mutual understanding of values can be developed. Chapter 7 focusses on digital health and the importance of a co-design mindset in developing or adopting technology.

The final two chapters address how integrated care programmes can be implemented and evaluated. The inter-professional and cross-organisational dynamics are identified as key challenges of implementing integrated care with Kotter’s framework then used to guide the reader through a positive change process. The book concludes with four approaches to evaluate integrated care based on the logic model of the integrated care programme and the purpose of the evaluation.

The book provides an accessible and informative introduction to the rationale, concepts and implementation of integrated care. Relevant academic theories and research are drawn upon and sucessfully connected with application in practice. The chapters not only cover the traditional building blocks of integrated care but add in new thinking and opportunities, such as leadership, social dimensions and use of technology. The authors bring credibity in their respective fields with their international spread ensuring that learning goes beyond a particular region or health system. The chapters are of good length – long enough to provide a good level of insights, but short enough that a busy reader should not feel intimidated.

Perhaps reflecting its origins, the book is more orientated to those working in clinical health care settings rather than those working within social services and / or the community sector but will still have relevance for all. It would have been helpful to consider issues relating to the frontline workforce such as new job roles, team development and training and education. The inclusion of practical case studies in all chapters would have brought further life to the theory and research and a more obvious theming of the order of chapters would have enabled further connection between the chapters. These are though minor critiques – the book is a helpful and well written collection.

This book is highly recommended to health care managers, professionals and researchers who are looking for an informed introduction to the field of integrated care. It rightly avoids the temptation to provide straightforward answers and tick box guidance on how to better integrate care. Instead it responds to the complexity of the task by providing informed insights on how to understand the context and challenges and work with stakeholders to develop impactful and sustained solutions.

